# Predictive values of ultrasonic diaphragm thickening fraction combined with integrative weaning index in weaning patients with mechanical ventilation: a retrospective study

**DOI:** 10.1186/s13019-024-02544-x

**Published:** 2024-02-06

**Authors:** Hai Lin, Mingming Yao, Ziwen Qin, Shilin Fu, Hongyou Wang

**Affiliations:** Emergency Department, Maanshan People’s Hospital, No. 45 Hubei Road, Ma’anshan, Anhui 243000 China

**Keywords:** Diaphragm thickening fraction, Integrative weaning index, Mechanical ventilation, Weaning, Ultrasound

## Abstract

**Objective:**

We aimed to explore the predictive values of ultrasonic diaphragm thickening fraction (DTF) combined with integrative weaning index (IWI) in weaning patients with mechanical ventilation.

**Methods:**

Patients with mechanical ventilation who received oral endotracheal intubation from September 2020 to September 2021 were included in this retrospective study. Before the start of the spontaneous breathing test (SBT), IWI was calculated according to the blood gas analysis parameters and parameters read in volume control mode. After the start of SBT, DTF was calculated according to the end-expiratory thickness and end-inspiratory thickness of the right diaphragm. The receiver operating curve (ROC) was used to evaluate the predictive value of DTF and IWI for successful weaning, and the sensitivity and specificity were calculated according to the best critical value.

**Results:**

The sensitivity, specificity, and best cutoff value of DTF to predict successful weaning was 0.772, 0.727, and 0.293, respectively, and the area under the curve (AUC) was 0.72 (95%CI 0.59–0.86, *p* = 0.003). The sensitivity, specificity, and best cutoff value of IWI to predict successful weaning was 0.614, 0.909, 53.00, respectively, and AUC was 0.82 (95%CI 0.72–0.91, *p* < 0.001). The sensitivity, specificity, and best cutoff value of the combination of DTF and IWI to predict successful weaning was 0.614, 0.909, 17.848, respectively, and AUC was 0.84 (95%CI 0.75–0.93, *p* < 0.001).

**Conclusion:**

DTF and IWI can guide the selection of weaning, while DTF combined with IWI can improve the effect of weaning prediction and provide support for patients’ weaning safety.

## Introduction

Invasive mechanical ventilation is now one of the most commonly used short-term life support techniques worldwide [1]. With the development of invasive mechanical ventilation techniques, physicians have progressively mastered the clinical use timing of invasive mechanical ventilation. However, the choice of weaning time remains a challenge. Applying clinical experience or a single parameter to determine the weaning timing can be risky, and either premature or delayed weaning can harm clinical outcomes [2].

A spontaneous breathing test (SBT) is often used to predict the likelihood of successful weaning, which is a standardized and clinically accepted procedure for weaning. Studies have shown that approximately 30% of mechanically ventilated patients fail their first SBT test [3], and approximately 11.6% who pass the SBT test for at least 30 min may still fail before the test is extended to 120 min [4]. Even if they pass the SBT trial and are extubated, approximately 15% of mechanically ventilated patients are still at risk of reintubation within 48 h. Repeated extubation and delayed weaning can lead to increased morbidity and mortality, longer ICU stays and longer hospital stays [5]. Due to the many uncertainties in the process of weaning from ventilator, some patients are at risk of reintubation or even death, even if a standardized process of weaning from ventilator is developed and implemented according to the SBT trial. Therefore, finding and applying clinical parameters to guide weaning and predict outcomes may enable timely and successful weaning of mechanical ventilation.

Diaphragm thickening fraction (DTF) has been shown to correlate with lung volume. DTF ≥ 27.9% is used to predict successful weaning with a sensitivity of 98.3%, a specificity of 62.1%, and an area under the curve (AUC) of 0.873 [6]. The integrated weaning index (IWI) is first proposed in a large prospective study by Sergio in 2009, and the results show that IWI is a good predictor of weaning, with an AUC of 0.96, a sensitivity of 97%, and a specificity of 94% [7]. However, both DTF and IWI have certain limitations in predicting the outcomes of the weaning. Therefore, we aimed to explore the predictive values of DTF combined with IWI in weaning patients with mechanical ventilation.

## Patients and methods

### Patients

Mechanically ventilated patients admitted to the Intensive Care Unit of the Ma’anshan People’s Hospital from March 2021 to March 2022 were included in this retrospective study that was adherence to STROBE guidelines. The study was approved by the Ethics Committee of Ma’anshan People’s Hospital. The informed consent was obtained. Patients were divided into a successful group for successful weaning and a failure group for failed weaning based on their weaning outcome. Inclusion criteria: (1) age ≥ 18 years; (2) patient’s etiology controlled or removed; (3) duration of mechanical ventilation ≥ 24 h; (4) no deterioration in neurological Glasgow Coma Scale (GCS); (5) hemodynamic stability; (6) oxygenation index > 150 mmHg, positive end-expiratory pressure < 5–8 cmH_2_O; (7) some cough reflex; (8) successful passage of SBT test. Exclusion criteria: (1) patients with severe myopathy; (2) pregnant women; (3) patients with tracheotomy; (4) patients with spinal cord injury above the T8 plane; (5) patients with diaphragm injury. Criteria for successful weaning: maintenance of spontaneous breathing for 48 h after extubation without reintubation; criteria for failure to withdraw: need for reintubation for mechanical ventilation within 48 h after extubation or death of the patient.

### SBT procedure

The range of SBT: (1) the cause is controlled or removed; (2) hemodynamic stability; (3) no new potential serious lesions; (4) PEEP < 5 ~ 8 cmH_2_O, PaO_2_/FiO_2_ > 150 mmHg, FiO_2_ ≤ 40%, PH ≥ 7.25; (5) Ability of spontaneous breathing and airway clearance. SBT was performed using the T-tube method, with a test time of 30 to 120 min. The SBT will fail and be terminated if any of the following conditions occurred and the duration was > 5 min. (1) Deterioration of alveolar gas exchange function (reference indicators: SPO_2_ ≤ 85-90%; PaO_2_ ≤ 50–60 mmHg; PH ≤ 7.32; increase in PaCO_2_ ≥ 10 mmHg); (2) deterioration of hemodynamic status (reference indicators: HR ≥ 120–140 beats/min, or increase > 20%; SBP < 90 mmHg or > 180–200 mmHg, or > 20% change; an increased dose of vasoactive drugs); (3) deterioration in respiratory form (reference indicators: RR ≥ 35 beats/min, or > 50% change); (4) marked deterioration in mental status (reference symptoms: drowsiness, coma, agitation, anxiety, etc.); (5) marked subjective discomfort; (6) marked sweating; (7) marked increase in respiratory work (reference symptoms: auxiliary respiratory muscles involved in breathing, thoracoabdominal paradoxical breathing).

### IWI measurement

In ICU, different ventilator modes are selected according to the patient’s condition, such as the commonly used volume control mode. However, for patients who are about to enter the offline process, the ICU will adjust the ventilator mode to PSV mode at least 1 h in advance, and suspend the patient’s sedative and analgesic drugs, so that the patient can breathe spontaneously and participate in the whole offline process. First, conduct a blood gas analysis to obtain arterial oxygen saturation (SaO_2_). Second, platform pressure (Pplat), tidal volume (Vt), positive end-expiratory pressure (PEEP), and respiratory rate (f) were read in volume control mode. Third, calculate lung static compliance (Cst, rs). Cst, rs = Vt/(Ppalt-PEEP, ml/cmH_2_O). Fourth, calculate the respiratory shallow fast index (RSBI). RSBI = f/V_t_ (bpm/L). Finally, according to the formula: IWI = Cst, rs × SaO_2_ / (f/Vt), the IWI value (ml/cmH_2_O/(bpm/L)) was obtained.

### DTF measurement

**Fig. 1:**
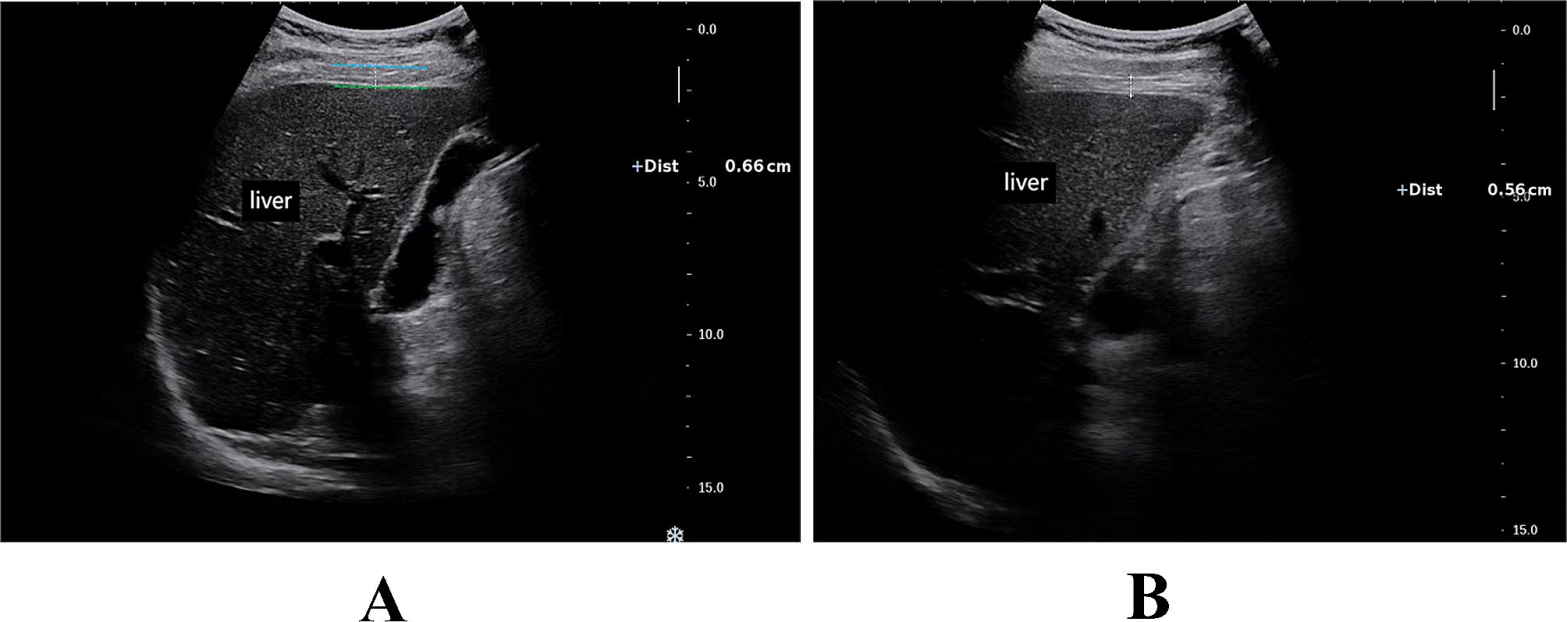
Ultrasound images of end-inspiratory diaphragm thickness and end-expiratory diaphragm thickness. (**A**) Diaphragm thickness at the end of inspiration (Dtei); (**B**) Diaphragm thickness at the end of exhalation (Dtee). The green dashed line represents the visceral pleura and the blue dashed line represents the parietal pleura (in Fig. 1A). The parallel high echo light band near the probe side is the parietal pleura, while the parallel high echo light band far from the probe side is the visceral pleura. The low echo structure located between the parietal pleura and visceral pleura is the diaphragm (in Fig. 1B)

After the start of SBT, with the patient’s head elevated at 30° in the absence of contraindications, diaphragm thickness was measured and DTF was calculated using a portable bedside ultrasonograph (EDGE II; Sonosite) between 5 and 30 min after the start of the T-tube. Using a 3.5-5 MHz convex array probe placed in the 8th to 10th rib space in the anterior or mid-axillary line, the ultrasound mode was used to show the structures attached to the foot of the diaphragm in the right lower chest wall, using the liver as the acoustic window. The tissue structures in this area were divided into three layers: the parallel hyperechoic band near the probe side was the mural pleura, the parallel hyperechoic band away from the probe side was the peritoneum, and the hypoechoic structures located between the mural pleura and the peritoneum was the diaphragm. The thickness of the diaphragm was measured and recorded at the end of inspiration and the end of respiration, using the M ultrasound mode, with the measurement line perpendicular to the diaphragm (Fig. 1). The intervals were 5 min and the measurements were repeated 2 times and averaged. DTF = (end-inspiratory diaphragm thickness - end-expiratory diaphragm thickness) / end-expiratory diaphragm thickness. The DTFs were also tested for consistency by the Bland-Altman diagram.

### Observation indexes

(1) General clinical indicators were collected, including gender, age, acute physiology, and chronic health evaluation II (APACHE II score), oxygenation index; (2) DTF; (3) IWI.

### Statistical analysis

Data were processed using SPSS 26.0 statistical software. The measurement data were expressed as mean ± standard deviation (SD). Normally distributed data were compared using the independent samples *t*-test. Non-normal distribution data were expressed using median M and quartiles M (P25, P75), and the rank sum test was used for comparison between the two groups. Counting data were expressed as frequency (percentage) [n (%)], and the chi-square test was used for comparison between the two groups. Receiver operating curve (ROC) curves were plotted to evaluate the values of DTF, IWI, and DTF combined with IWI in measuring the success of weaning. Sensitivity, specificity, and optimal cut-off values were calculated. The optimal cut-off value was the value taken at the maximum of the “Jorden index”, i.e. the cut-off value at the maximum of sensitivity + specificity − 1, and the area under the curve (AUC) was calculated. *p* < 0.05 was considered a statistically significant difference.

## Results

### General clinical characteristics

A total of 79 patients were included in the study. The sample size was an unplanned recruitment result for the number. Of these, 59 (75%) were male and 20 (25%) were female. The reasons for the patients’ mechanical ventilation were shown in Table 1. The general conditions, clinical characteristics, and diaphragmatic ultrasound findings of the two groups were shown in Table 2. There were 22 (28%) patients who failed weanings and 57 (72%) had successful weanings. The differences in gender and age between the two groups were not statistically significant (*p* > 0.05). APACHE II scores in the failure group were increased in comparison with the success group (*p* < 0.001]). The oxygenation index in the failure group was decreased in comparison with the success group (*p* < 0.001).

**Table 1:** Disease diagnosis and number of cases

Disease diagnosis	Cases (n)	%
Cerebrovascular accident	16	20.25
Multiple injuries, fractures	14	17.72
Tumors	12	15.19
Craniocerebral injury	11	13.92
Peritonitis	10	12.66
Perforation and bleeding of the digestive tract	5	6.33
Septic shock	4	5.06
Closed abdominal injury	2	2.53
Pneumonia	1	1.27
Chronic obstructive pulmonary disease	1	1.27
Myocardial infarction	1	1.27
Heart failure	1	1.27
After cardiopulmonary resuscitation	1	1.27
Total	79	-

**Table 2:** Comparison of general clinical characteristics and diaphragm ultrasound findings between the two groups

Items	Failure group (*n* = 22)	Success group (*n* = 57)		Statistics		*p*
General clinical characteristics						
Gender (male, n,%)	19(86.3)	40(70.2)		2.200		0.138
Age (years)	68(56,78)	68(54,77)		-0.103		0.918
APACHE2 scores	21(15,25)	12(8,17)		-4.182		< 0.001
Oxygenation index(mmHg)	239.535 ± 94.084	305.508 ± 114.127		2.411		0.018
SaO_2_(%)	97 ± 3.232	98.08 ± 2.52		1.522		0.132
Pplat (cmH_2_O)	23.890 ± 1.197	20.350 ± 0.971		-13.093		< 0.001
Vt (ml)	408.950 ± 19.118	456.170 ± 26.876		8.443		< 0.001
F (time/min)	28(25,35)	20(18.22)		-6.106		< 0.001
RSBI (bpm/L)	71.429(64.103,85.366)	44.315(40.523,48.837)		-6.350		< 0.001
Cst, rs (ml/cmH_2_O)	23.408 ± 1.560	30.094 ± 2.403		-6.176		< 0.001
IWI (ml/cmH_2_O/bpm/L)	37.613 ± 11.423	57.415 ± 17.773		5.846		< 0.001
Ultrasound indicators						
Dtei-1	0.348 ± 0.070	0.348 ± 0.069		3.236		0.002
Dtei-2	0.346 ± 0.067	0.401 ± 0.070		3.439		0.002
Dtee-1	0.274 ± 0.055	0.289 ± 0.047		1.135		0.308
Dtee-2	0.277 ± 0.052	0.286 ± 0.045		0.766		0.446
DTF-1	0.246 ± 0.026	0.385 ± 0.063		13.781		< 0.001
DTF-2	0.23 ± 0.035	0.395 ± 0.068		0.766		< 0.001
Average DTF	0.287 ± 0.081	0.350 ± 0.078		3.200		0.002

APACHE2, acute physiology and chronic health evaluation 2; SaO_2_, arterial oxygen saturation; Pplat, read platform pressure; V_t_, tidal volume; F, respiratory rate; RSBI, rapid shallow breathing index; Cst, static lung compliance; IWI, integrative weaning index; DTF, diaphragm thickening fraction; Dtei, diaphragm thicknesses; Dtee, diaphragmatic thicknesses

### Analysis of IWI

The successful and failed groups showed significant differences in terms of Pplat (23.89 ± 1.20 vs. 20.35 ± 0.97, *p* < 0.001), Vt (408.95 ± 19.12 vs. 456.17 ± 26.88, *p* < 0.001), f (28(25,35) vs. 20(18.22), *p* < 0.001), RSBI (71.43(64.10,85.37) vs. 44.32(40.52,48.84), *p* < 0.001), Cst, rs (23.41 ± 1.56 vs. 30.09 ± 2.40, *p* < 0.001), IWI (37.61 ± 11.42 vs. 57.42 ± 17.77, *p* < 0.001). However, there was no statistical difference in SaO_2_ (*p* = 0.132) between the two groups.

### Analysis of DTF

The 2 DTF measurements were analyzed using the consistency test (Bland-Altman diagram) method. The mean difference was 0.088, with 95% agreement limits ranging from 0.181 to 0.525. In the two measurements, only 2 (2/79, 2.5%) DTF-1 and 0 (0/79, 0%) points were outside the 95% consistency limit. The rest were within the 95% consistency limit. The consistency of the two measurements was high.

### Analysis of diaphragm ultrasound indicators

There were statistical differences between the success and failure groups in terms of two end-inspiratory diaphragm thicknesses (Dtei) (Dtei-1: *p* = 0.002; Dtei-2: *p* = 0.002), and mean diaphragm thickening fraction (DTF) (0.287 ± 0.081 vs. 0.350 ± 0.078, *p* = 0.002). There was no statistical difference between the success and failure groups in terms of Two end-expiratory diaphragmatic thicknesses (Dtee) (Dtee-1: *p* = 0.308; Dtee-2: *p* = 0.446).

### Predictive analysis of the weaning results

**Fig. 2:**
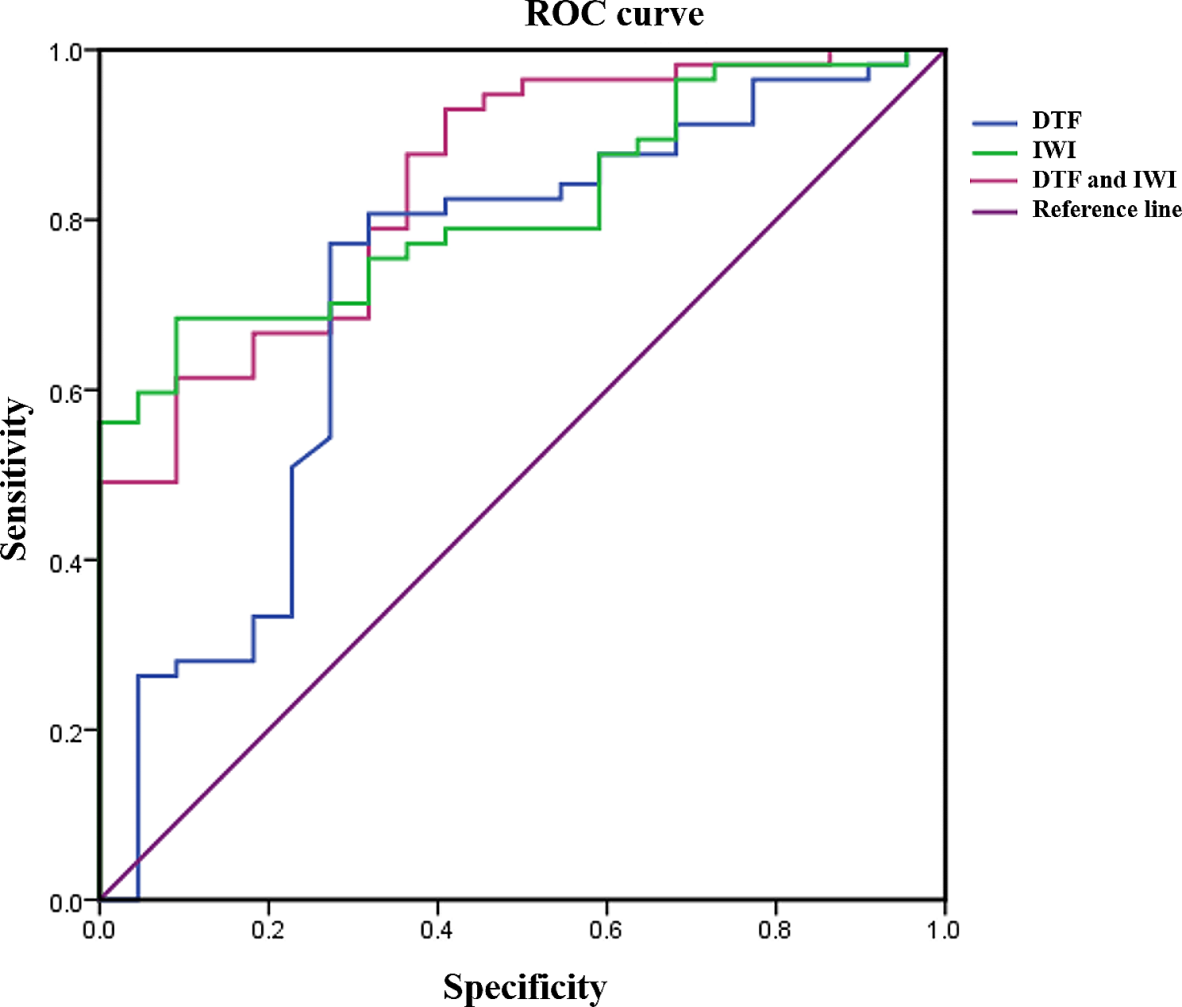
Predicted weaning efficiency by ROC curve. ROC, receiver operating curve

ROC analysis was shown in Fig. 2; Table 3. DTF refers to the thickening fraction of the diaphragm, the unit is %, and the cutoff value was 29.3%. The unit of IWI was ml/cmH_2_O/(bpm/L), and the cutoff value was 53.0 ml/cmH_2_O/(bpm/L). The sensitivity, specificity, and best cutoff value of DTF to predict successful weaning was 0.772, 0.727, and 0.293, respectively, and AUC was 0.72 (95%CI 0.59–0.86, *p* = 0.003). The sensitivity, specificity, and best cutoff value of IWI to predict successful weaning was 0.614, 0.909, 53.00, respectively, and AUC was 0.82 (95%CI 0.72–0.91, *p* < 0.001). The sensitivity, specificity, and best cutoff value of the combination of DTF and IWI to predict successful weaning was 0.614, 0.909, 17.848, respectively, and AUC was 0.84 (95%CI 0.75–0.93, *p* < 0.001).

**Table 3:** Predictive efficacy of DTF, IWI, and combined application on withdrawal

Items	Sensitivity	Specificity	Best cutoff value	AUC(95%CI)	*p*
DTF	0.772	0.727	0.293	0.72(0.59 ~ 0.86)	0.003
IWI	0.684	0.909	53.000	0.82(0.72 ~ 0.91)	< 0.001
DTF and IWI	0.614	0.909	17.848	0.84(0.75 ~ 0.93)	< 0.001

IWI, integrative weaning index; DTF, diaphragm thickening fraction

## Discussion

In this study, we found that DTF and IWI can guide the selection of weaning, while DTF combined with IWI can improve the effect of weaning prediction and provide support for patients’ weaning safety.

In clinical care, patients with respiratory failure often require ventilator-assisted breathing. Mechanical ventilation is the most widely used form of respiratory support in clinical practice [1]. It allows patients with respiratory failure to change from shallow fast breathing to normal breathing without increasing the work done by breathing and significantly improves alveolar ventilation [8, 9]. However, mechanical stimulation during mechanical ventilation can lead to a more severe inflammatory response in the patient’s organism [10, 11]. In addition, prolonged mechanical ventilation can lead to progressive multi-organ damage and consequent multi-organ failure, increasing the risk of death [12]. Therefore, the ventilator should be withdrawn promptly when the underlying reasons for the need for mechanical ventilation have been sufficiently ameliorated. Although the success rate of weaning is high, there are still some patients who are unable to pass SBT or who need to be reintubated within a short period. Therefore, the successful weaning of mechanically ventilated patients in respiratory failure remains an important clinical challenge.

Failure to withdraw invasive mechanical ventilation is associated with the clinical prognosis [13]. Weaning failure has been defined as the interruption of SBT due to clinical intolerance, the patient’s need for non-invasive or invasive mechanical ventilation after weaning, or the patient’s death within 48 h of being taken off the machine [14]. Patients who failed SBT were not included in the sample for this study because even with SBT trials, between 15% [4] and 32% [15] of patients still experienced weaning failure. And half of the patients who failed weaning died during their ICU stay [16]. Due to the high number of pathological, individualized, and medically-derived factors that influence evacuation outcomes, exploring the parameters associated with patients who succeed or fail evacuation through a standardized SBT process may be more beneficial in improving evacuation success rates.

With the application and popularity of bedside ultrasound technology, it is known as a visual “stethoscope” due to its rapid, non-invasive, reproducible, and intuitive advantages [17]. The application of bedside ultrasound in the weaning of invasive mechanical ventilation includes diaphragm function monitoring, lung ultrasound, volume responsiveness assessment, and cardiac ultrasound indicators. Bedside ultrasound is a good aid in determining the pathophysiological status of patients and the reasons for weaning failure. An important indicator of diaphragm function is the diaphragm thickening fraction (DTF) [18].

A meta-analysis [19] that included 60 studies, including 4382 patients, showed that DTF had the highest predictive power for weaning failure, with a combined sensitivity of 0.76 (95% CI 0.67–0.83) and specificity of 0.86 (95% CI: 0.78–0.92). Another meta-analysis [20], which included 16 studies including 816 patients, showed that DTF had a combined sensitivity of 0.70 (95% CI 0.57–0.80), specificity of 0.84 (95% CI 0.73–0.91) and AUC of 0.82 (95% CI 0.78–0.85) for predicting weaning outcomes. This study was consistent with the previous studies in terms of sensitivity and AUC, but had a slightly lower specificity and was prone to false negative results. The reason for this may be that weaning is also influenced by non-diaphragm-related factors. For example, when the primary morbidity is controlled and the lung compliance and airway environment are good, the diaphragm is still able to produce adequate ventilation when there is little change in diaphragmatic thickening. Therefore, further research is needed to apply a uniform definition of deconditioning and uniformly implemented process measures in a subgroup of critically ill patients. Incorporating individualized patient differences reduces the heterogeneity of the study to improve the accuracy of diaphragmatic ultrasound-guided deconditioning.

IWI is essentially a composite parameter that includes static pulmonary compliance (Cst, rs), arterial oxygen saturation (SaO_2_), and the rapid shallow breathing index (RSBI). According to the formula, IWI is positively correlated with Cst, rs, and SaO_2_ and negatively correlated with RSBI. In this study, IWI was found to be less sensitive than DTF in predicting weaning success, but with higher specificity. Applying IWI as a weaning guide may be effective in avoiding the damage caused by premature weaning. Previous studies have found that using IWI > 45.70 mL/cmH_2_O/bpm/L as the threshold for predicting successful weaning has a sensitivity of 0.91, a specificity of 0.83, and an AUC of 0.91 [21]. Although this study did not indicate a higher sensitivity, the specificity (0.90), AUC (0.84), and best cut-off value (53.00) were consistent with previous studies.

The factors affecting weaning are as follows: (1) Airway and lung dysfunction. Increased airway resistance, decreased respiratory compliance, and impaired gas exchange increase respiratory work, leading to weaning failure. (2) Brain dysfunction. Brain dysfunction in patients with difficult weaning is mainly related to delirium. Studies have shown that impaired cognitive function increases the risk of extubation failure by more than 4 times. Other psychological disorders, such as anxiety and depression, can affect successful weaning. Sleep interruption often occurs in ICU patients, but there is no data showing the effect of sleep structure disorder on weaning. (3) Cardiac dysfunction. (4) Diaphragm / respiratory muscle function. SBT offline test will produce acute load on inspiratory muscles. It has been studied that patients with mechanical ventilation for 18–96 h have severe muscle fiber atrophy in the diaphragm, accompanied by activation of the proteolytic ubiquitin-proteasome pathway. At the same time, clinically relevant diaphragmatic dysfunction is sometimes caused by phrenic nerve damage, such as critical illness polyneuropathy (CIP). When considering inspiratory muscles as a cause of weaning failure, it is common to consider whether there is a lesion between the conduction pathway of the respiratory center and the contractile protein.

There are several weaning parameters in common use today. The aim of using them in combination is to better predict the weaning outcome and to avoid damage to the patient from delayed or premature weanings. Ultrasound diaphragmatic examinations provide clinicians with visual images of diaphragmatic movements and can quantify the magnitude of changes in diaphragmatic movements, thus indirectly reflecting the patient’s ability to drive breathing. The composite deconditioning index, on the other hand, is a good indicator of lung tissue elasticity as it involves parameters such as SaO_2_, lung static compliance, respiratory rate, and tidal volume. Combining the two advantages can therefore improve the accuracy of weaning prediction.

In this study, weaning outcomes failed in three patients with myocardial infarction, heart failure, or CPR. In two of these patients, IWI was inaccurate in predicting the weaning outcomes. This suggests that the use of cardiac function-related parameters in combination to predict the weaning outcome may be beneficial when a serious cardiogenic cause is present. It may be more reasonable to apply different parameters to predict weaning outcomes between cases, but this conclusion needs to be further confirmed in a larger sample of studies. This also demonstrates the complexity of the factors influencing the different clinical outcomes after weaning. Therefore, within the context of a standardized weaning process, experienced clinicians should be encouraged to develop individualized weaning protocols using ultrasound and ventilator parameters, taking into account the patient’s condition.

There are also some limitations in this study. First of all, this study is a single-center retrospective study, and the strength of causality argument is limited. Secondly, the sample size is relatively small, and there is a certain selection bias, so it is necessary to be cautious when extrapolating conclusions. In the future, large-sample, multi-center, longitudinal follow-up studies are still needed to further explore the influencing factors of weaning success in patients with mechanical ventilation.

## Conclusion

In conclusion, DTF and IWI were good predictors of weaning outcomes in mechanically ventilated patients who passed the SBT trial. DTF combined with IWI was beneficial in improving weaning prediction and supporting the safe weaning of patients.

## Data Availability

The datasets used and/or analyzed during the current study are available from the corresponding author on reasonable request.

## Abbreviations

AUC: Area under the curve
Cst: Static lung compliance
DTF: Diaphragm thickening fraction
F: Respiratory rate
HR: Heart rate
IWI: Integrative weaning index
PaO_2_: Arterial partial pressure of oxygen
PaCO_2_: Arterial carbon dioxide pressure
PEEP: Positive end-expiratory pressure
Pplat: Read platform pressure
ROC: Receiver operating curve
RSBI: Rapid shallow breathing index
SaO_2_: Arterial oxygen saturation
SBP: Systolic blood pressure
SBT: Spontaneous breathing test
V_t_: Tidal volume

## References

[1] Pham T, Brochard LJ, Slutsky AS (2017). Mechanical ventilation: state of the art. Mayo Clin Proc.

[2] Baptistella AR, Sarmento FJ, da Silva KR (2018). Predictive factors of weaning from mechanical ventilation and extubation outcome: a systematic review. J Crit Care.

[3] Esteban A, Frutos F, Tobin MJ (1995). A comparison of four methods of weaning patients from mechanical ventilation. N Engl J Med.

[4] Liang G, Liu T, Zeng Y (2018). Characteristics of subjects who failed a 120-Minute spontaneous breathing trial. Respir Care.

[5] McConville JF, Kress JP (2012). Weaning patients from the ventilator. N Engl J Med.

[6] Ning Lin Q, Huang H, Zhang (2019). Application of diaphragmatic ultrasound in mechanical ventilation patient weaning. Chin J Respiratory Crit Care.

[7] Nemer SN, Barbas CS, Caldeira JB (2009). A new integrative weaning index of discontinuation from mechanical ventilation. Crit Care.

[8] McGuinness G, Zhan C, Rosenberg N (2020). Increased incidence of Barotrauma in patients with COVID-19 on invasive mechanical ventilation. Radiology.

[9] Kim HJ, Choi SM, Lee J (2018). Respiratory virus of severe pneumonia in South Korea: prevalence and clinical implications. PLoS ONE.

[10] Uda K, Koyama-Wakai C, Shoji K (2018). WU Polyomavirus detected in children with severe respiratory failure. J Clin Virol.

[11] Azoulay E, Russell L, Van de Louw A (2020). Diagnosis of severe respiratory infections in immunocompromised patients. Intensive Care Med.

[12] Goligher EC, Dres M, Fan E (2018). Mechanical ventilation-induced Diaphragm Atrophy strongly impacts Clinical outcomes. Am J Respir Crit Care Med.

[13] Thille AW, Richard JCM, Brochard L (2013). The decision to Extubate in the Intensive Care Unit. Am J Respir Crit Care Med.

[14] Palkar A, Mayo P, Singh K (2018). Serial diaphragm Ultrasonography to predict successful discontinuation of mechanical ventilation. Lung.

[15] Zein H, Baratloo A, Negida A (2016). Ventilator weaning and spontaneous breathing trials;an Educational Review. Emergency.

[16] Jung B, Moury PH, Mahul M (2016). Diaphragmatic dysfunction in patients with ICU-acquired weakness and its impact on extubation failure. Intensive Care Med.

[17] Expert Consensus Group on the Clinical Application of Bedside (2016). Ultrasound in Acute and critical illness Expert consensus on the clinical application of bedside ultrasound in critically ill patients. Chin J Emerg Med.

[18] Chen D, Shi J, Chen X (2021). Progress in the application of bedside ultrasound in predicting difficulty offline in patients with invasive mechanical ventilation. Chin J Emerg Med.

[19] Medrinal C, Combret Y, Hilfiker R (2020). ICU outcomes can be predicted by noninvasive muscle evaluation: a meta-analysis. Eur Respir J.

[20] Le Neindre A, Philippart F, Luperto M (2021). Diagnostic accuracy of diaphragm ultrasound to predict weaning outcome: a systematic review and meta-analysis. Int J Nurs Stud.

[21] Huo Y, Guo S, Zhang K, Zhang T (2020). A clinical study on the ability of the integrative weaning index to predict weaning from mechanical ventilation. Ann Palliat Med.

